# Inhaling Difluoroethane Computer Cleaner Resulting in Acute Kidney Injury and Chronic Kidney Disease

**DOI:** 10.1155/2018/4627890

**Published:** 2018-06-07

**Authors:** Kristen Calhoun, Laura Wattenbarger, Ethan Burns, Courtney Hatcher, Amol Patel, Manjulatha Badam, Abdul-Jabbar Khan

**Affiliations:** ^1^Texas A&M College of Medicine, The Methodist Hospital at Houston, 6565 Fannin Street, West Pavilion, Houston, TX 77030, USA; ^2^Houston Methodist Hospital, 6550 Fannin Street, Suite 1101, Smith Tower, Houston, TX 77030, USA

## Abstract

Difluoroethane is the active ingredient in various computer cleaners and is increasingly abused by teenagers due to its ease of access, quick onset of euphoric effects, and lack of detectability on current urine drug screens. The substance has detrimental effects on various organ systems; however, its effects on the kidneys remain largely unreported. The following case report adds new information to the developing topic of acute kidney injury in patients abusing difluoroethane inhalants. In addition, it is one of the first to show a possible relationship between prolonged difluoroethane abuse and the development of chronic kidney disease in the absence of other predisposing risk factors.

## 1. Introduction

Difluoroethane (DFE), the active ingredient in aerosol sprays such as “Dust Off” computer cleaner, is becoming a popular substance of abuse, particularly among teenagers [1]. Due to its ease of access and availability, the incidence and prevalence of difluoroethane abuse are increasing [2]. Approximately 11% of high school students report experimenting at least once with inhalants such as DFE, paint thinner, or nitrous oxide (Table 1) [1]. There is an expanding breadth of knowledge of the potential detrimental effects associated with DFE including acute kidney injury (AKI), angioedema, frostbite, cardiomyopathy, skeletal fluorosis, and fatal arrhythmias occurring within minutes of use (Table 1) [3–9]. Few case reports have described the effects of difluoroethane on kidney function, and there have not yet been any cases describing the link between DFE and chronic kidney disease (CKD). The following case describes a patient presenting with DFE toxicity leading to both AKI and CKD.

## 2. Case Presentation

A 32-year-old Caucasian male with a known history of depression presented to the emergency department accompanied by police after a violent outburst following prolonged DFE abuse during a suicide attempt. On admission, he was emotionally labile and had contusions on his left shoulder and upper extremities due to a physical altercation with police. He was initially confused and verbally abusive, but within six hours he was oriented, cooperative, and able to provide a reliable history.

The patient stated that he had huffed keyboard cleaner several days per week for the past year; however, the frequency had increased over the last month to several times daily. Other than depression, the patient had no other known medical problems. He denied any personal or family history of kidney disease. The patient denied drinking alcohol, and he reported smoking one-half pack of cigarettes per day for the previous two years. He denied any other substance abuse except for DFE. He reported no use of nonsteroidal anti-inflammatory (NSAID) medications. During this episode of difluoroethane abuse, he had no loss of consciousness, but experienced frightening visual and auditory hallucinations as well as anxiety that persisted even after returning to his self-reported baseline mental status.

Initial labs revealed a WBC count of 21,000 with polymorphonuclear predominance of 81%, an elevated creatinine of 1.5mg/dL with no known baseline, BUN of 10mg/dL, GFR of 54 mL/min/1.73 m^2^, lactic acidosis, creatine kinase of 350 U/L, and a carboxyhemoglobin level of 3.1%. Urinalysis was significant for 3+ proteinuria, moderate blood, 14 RBCs, 3 WBCs, and 3 hyaline casts. He had a negative urine drug screen.

**Figure 1:**
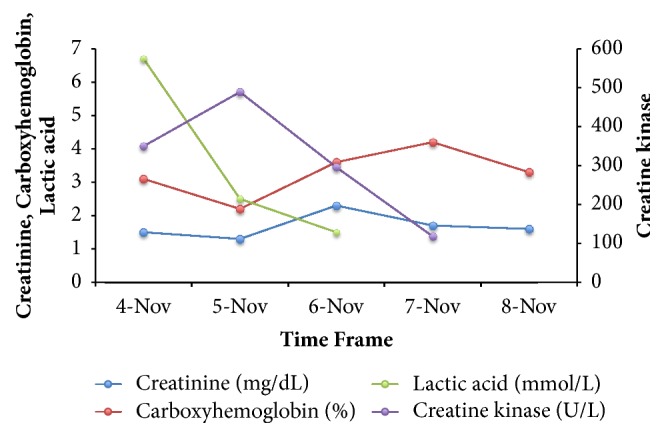
Lab trends during patient's hospital stay. The left axis displays creatinine, carboxyhemoglobin, and lactic acid and the right axis displays creatine kinase.

The patient received single renally adjusted doses of Vancomycin and Piperacillin-Tazobactam, intravenous 0.9% saline, and oxygen by nasal cannula while in the emergency department prior to admission. Over the next 24 hours, he received intravenous fluids and oxygen with subsequent normalization of lactic acid, creatine kinase, and leukocyte count. His creatinine down trended to 1.3mg/dL. However, 36 hours into admission, his serum creatinine inexplicably rose to 2.3 with a rise in carboxyhemoglobin to 3.6%, while his urine output remained stable (Figure 1). Repeat urinalysis showed a small amount of blood with RBC of 2, negative proteinuria, and no evidence of infection.

**Figure 2:**
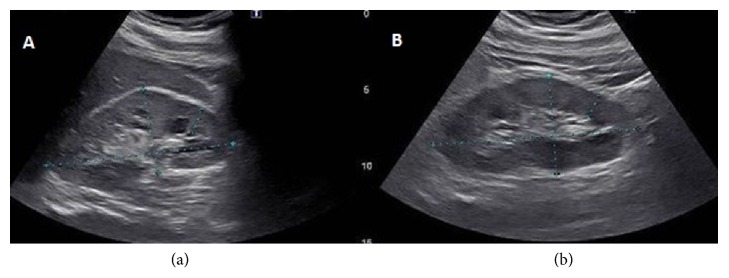
Renal ultrasound. (a) Sagittal section of the right kidney demonstrating increased echogenicity. The right kidney measures 12.3 x 5.5 x 5.9 centimeters. (b) Sagittal section of the left kidney demonstrating increased echogenicity (blue line). The left kidney measures 13.4 x 6.4 x 5.2 centimeters.

His normal saline infusion was transitioned to bicarbonate with saline, but was discontinued after the patient developed pruritus. Ultimately, he was transitioned to normal saline at 75 ml/hour. A renal ultrasound showed increased echogenicity of both kidneys consistent with medical renal disease, without change in size, atrophy, or cystic lesions (Figure 2). Thus, it is likely that the patient had some degree of chronic kidney disease (CKD) prior to admission. For the next three days, he continued to receive intravenous normal saline with subsequent improvement of creatinine and normalization of his urinalysis and was discharged with a creatinine of 1.6.

## 3. Discussion

The concentration of DFE in the brain rises rapidly after inhalation leading to euphoria, but the levels also decrease in the brain within minutes (Table 1). Since the kidneys are highly perfused organs, it is possible that the patient's AKI and CKD are related to high concentrations of DFE deposition. Rat models have demonstrated that kidneys may be susceptible to accumulation of DFE, and theorized aldehydic metabolites (Table 1) could possibly predispose the kidneys to DFE toxicity. However, toxicity to date has not been reported in chronic dosing studies [10]. Avella et al. demonstrated an overall renal uptake of 0.32% out of 4% total uptake of the administered dose in rats exposed to 30 seconds of difluoroethane [10]. They also showed that difluoroethane concentrations were highest in the kidneys out of all tissues measured at 8 minutes, suggesting kidneys may be susceptible to DFE burden [10]. Keller et al. demonstrated the presence of difluoroethane metabolite accumulation in the kidneys in rat models, but did not look at where the accumulation specifically occurred within the kidney or for how long [11]. Additionally, there was no long-term follow-up to determine whether or not the DFE byproduct deposition leads to a chronic direct nephrotoxic effect.

The patient's initial AKI appeared prerenal with a urinary FeNa of 0.5% and FeUrea of 37%. While his initial AKI on admission may have been in part due to rhabdomyolysis, the increase in creatinine after fluid resuscitation and normalization of creatine kinase is suggestive of another mechanism, such as a delayed ischemic event or a delayed accumulation of potentially nephrotoxic metabolites. Interestingly, his carboxyhemoglobin increased with the creatinine in the absence of smoke exposure. The reason behind this is not known, as this is not a known metabolite of DFE. Repeat urinalysis showed no casts, making direct tubular injury less likely.

Two case reports have described AKI in the setting of DFE use. Both patients were males in their thirties who developed AKI after loss of consciousness due to DFE abuse [3, 12]. Unlike this case report with delayed AKI, those patients presented with kidney injury on admission that resolved with IV fluids and thus had no evidence of chronic renal damage. While one report postulated the AKI to be secondary to dehydration and the other to be secondary to rhabdomyolysis or hypoperfusion, both mentioned the possibility of a direct toxic effect of difluoroethane [3, 12]. Our case is unique in that despite an initial improvement of creatinine from resolution of rhabdomyolysis and aggressive hydration, the patient's kidney function declined again, suggesting the possibility of delayed toxicity.

This patient's renal ultrasound findings were consistent with CKD, which is an unexpected finding in a nonobese 32-year-old without hypertension, diabetes, or NSAID use. He took no medication that would predispose him to CKD and had no known family history of CKD. With his lack of risk factors for CKD, it is possible that the ultrasound findings were secondary to chronic difluoroethane abuse. The mechanism by which difluoroethane is chronically nephrotoxic has yet to be discovered; however, based on the patient's labs and clinical progression, chronic accumulation involving toxic DFE metabolites is possible.

## 4. Conclusion

Few case reports have demonstrated AKI in the setting of acute DFE toxicity, with no existing reports of CKD in the setting of chronic DFE abuse. The pathophysiology of DFE induced nephrotoxicity is unknown and may be a combination of renal hypoperfusion, rhabdomyolysis, and chronic toxic accumulation of DFE metabolites. Further studies are needed to determine the exact mechanism by which difluoroethane is nephrotoxic.

## Figures and Tables

**Table 1:** Characteristics of difluoroethane [1, 10].

	**Difluoroethane Characteristics**
Incidence	11% of high school students have used an inhalant at least one time
Pathophysiology	Absorbed into blood via the alveoli, and distributed to end organs (the brain, heart, and kidneys) where metabolites may accumulate. It is hypothesized that metabolites are either exhaled or renally cleared. Increased GABA_A_ receptor affinity.
Symptoms	CNS depression and euphoria
Postulated Active Metabolites	Fluoroacetate, Fluorocitrate
Complications	Cardiomyopathy, Fatal Arrhythmias, Angioedema, Frostbite, Nephrotoxic

## Abbreviations

AKI: Acute kidney injury
CKD: Chronic kidney disease
DFE: Difluoroethane.
